# Fractal structures arising from interfacial instabilities in bio-oil atomization

**DOI:** 10.1038/s41598-020-80059-w

**Published:** 2021-01-11

**Authors:** Abbas Ghasemi, Sangsig Yun, Xianguo Li

**Affiliations:** 1 Department of Mechanical and Mechatronics Engineering, University of Waterloo, 200 University Avenue West, Waterloo, ON N2L 3G1 Canada; 2 Gas Turbine Laboratory, Aerospace Research Center, National Research Council, Ottawa, ON K1A 0R6 Canada

**Keywords:** Biofuels, Mechanical engineering, Fluid dynamics

## Abstract

The intriguing multi-scale fractal patterns ubiquitously observed in nature similarly emerge as fascinating structures in two-phase fluid flows of bio-oil breakup and atomization processes. High-resolution microscopy of the two-phase flows under 15 flow conditions (cases of different flow rates of the liquid and co-flowing air streams as well as different degrees of liquid preheating) reveal that the geometrical complexities evolve under the competing/combined action of the instability mechanisms such as Kelvin–Helmholtz, Rayleigh–Taylor and Rayleigh–Plateau leading into the transition from break-up to atomization. A thorough analysis of the higher order moments of statistics evaluated based on the probability density functions from 15,000 fractal dimension samples suggest that a single-value analysis is not sufficient to describe the complex reshaping mechanisms in two-phase flows. Consistently positive skewness of the statistics reveal the role of abrupt two-phase mechanisms such as liquid column rupture, ligament disintegration, liquid sheet bursting and droplet distortions in a hierarchical geometrical entanglement. Further, large kurtosis values at increased flow inertia are found associated with turbulence-induced intermittent geometrical reshaping. Interestingly, the proposed power-law correlation reveals that the global droplet size obtained from laser-diffraction measurements declines as the two-phase geometrical complexity increases.

## Introduction

In a pure mathematical point of view, fractals demonstrate a spatial self-similarity of geometrical patterns infinitely reproduced down to the smallest scales^1^. Remarkably, fractal structures ubiquitously appear in nature and often characterize certain physical phenomena such as complex patterns in butterflies’ wings^2^, liquid phases undergoing diffusion in microgravity^3^, fractal universality of galaxies^4^, DNA-based spatio-temporal analysis of the species and their genetic similarity/diversity^5^, soil particle size distribution^6^, evolutionary sensitivity of the animals sensory systems towards geometrically complex environments^7^, molecular complexity^8^, structural complexity of the human brain^9^, organisation of landscapes and river networks^10^, generation of fractal laser beams^11^, chemotherapy effects on the micro-vascular fractal structures^12^, aerosol aggregate in Jupiter’s atmosphere^13^, thermodynamic phase transition of glass^14^, turbulence-induced fractal patches affecting the phytoplankton ecology^15^, Alzheimer’s disease assessment through the complexity analysis of human cerebral cortex^16^ and lung tumour spatial growth over time^17,18^. As a revolutionary modern art technique, Jackson Pollock used dripping paint to create geometrically complex patterns^19^. His work has been analysed in terms of fractal characteristics emerging due to the interaction of falling liquid paint on the canvas surface^19^. While fractals are commonly observed as regular or irregular shapes in natural, biological and physical structures, they are not always perfectly self-similar, but very few studies consider their statistical distribution^20^.

In the area of thermofluids, and possibly in general, fractals can be associated with some source of instability leading into organized/chaotic patterns^21–23^, namely, Kelvin–Helmholtz (KH) instability in shear layers^24–28^ and viscous fingers^29,30^. Fractal complexities can be related to some important phenomena such as mixing and mass transport at the turbulent/non-turbulent interface of shear layers^31–33^ and boundary layers^34^ as well as turbulent particle agglomeration^35^. One of the most complex yet fascinating classes of fluid dynamics are the two-phase flows in which a competing effect from gravity^36^ and inertial forces against resistances arising from the differences in physical properties such as viscosity, surface tension and density produces a broad range of complex interfacial geometries. Such complexities arise from the onset of instability mechanisms such as Kelvin–Helmholtz (KH), Rayleigh–Taylor (RT) and Rayleigh–Plateau (RP) where initial linear growth of the perturbations transition into non-linear state and generate geometrically sophisticated structures in the form of liquid sheets, ligaments and distorted droplets of various length scales^37,38^. The KH instability sets in whenever a large velocity difference across an interface distorts the boundary between two layers of fluid and results in vortex roll-up^25^. As a result of the local acceleration at a section of a high-density fluid interface into the low-density medium, the RT instability is triggered^39^. The RP instability appears when nature tends to automatically minimize the interfacial energy through the act of surface tension^39^. In case of air-assisted atomization of liquid jets surrounded by high-momentum parallel air flow and subjected to shear layer instabilities^40^, atomization occurs in two successive steps. First, the gas/liquid interface experiences the KH type shear layer instability waves. In the second step, transient non-linear local accelerations on top of the primary KH waves trigger the RT instability. This results in the formation of ligaments that consequently break up into smaller droplets^41^. It should also be noted that the onset and growth of the instability mechanisms, which can be predicted by theory^42^, are highly affected by the mean velocity as well as turbulence levels introduced at the inlet. As a result of the combined effects from the mean flow as well as the turbulence intensity variations, determine the shape and length scale of the generated ligaments and droplets^43^. The morphology of primary and secondary gas/liquid interfacial waves, in terms of wavelength and amplitude, are the spatial manifestation of the instability mechanisms. Initiation and evolution of these surface instability waves are linked to the ligament and droplet formation^44,45^. In addition to experimental techniques, high-fidelity numerical methods such as direct numerical simulation (DNS) and large eddy simulation (LES) are able to resolve the smallest scales of turbulence affecting the atomization process. Detailed spatio-temporal analysis of the flow instabilities and their correlation with vortex dynamics reveal the complexities of break-up and atomization^46,47^.

Depending on the flow regime, ambient conditions and thermo-physical properties, the liquid break-up process may cascade down to the smallest possible length scales in fully atomized mode^48–50^. In addition to fundamental significance, two-phase spray dynamics play important role in variety of applications. Particular attention is increasing towards the implementation of bio-oils as viable renewable alternative fuels to mitigate the existing global threats against the climate due to elevated greenhouse gas (GHG) emissions^51–53^. As conventional fossil fuels eventually run out, they can be replaced by the carbon neutral biofuels produced from the abundantly available biomaterials in some remote communities and contribute to reduced transportation cost and emissions^54–56^. Optimized performance of bio-oil sprays in novel power generation applications or upgraded existing systems such as alternative aviation fuels^57^, internal combustion engines^58^ and gas turbines^59–61^ are limited by our lack of understanding on their peculiar two-phase behaviour. Due to the complex physiochemical properties of pyrolysis oils, resulting in their resistance to break-up and atomization^62–64^, the current fundamental knowledge of their underlying two-phase physics need to be improved.

Very few studies have explored the fractal characteristics of the liquid break-up leading to the spray formation^65,66^, mainly due to the intensive computations involved in the analysis of high-resolution images. However, the remarkable random and irregular geometrical structures of the liquid break-up demand extensive fractal analysis. Therefore, the present study is motivated for a comprehensive statistical analysis of the fractal complexities for a broad range of bio-oil break-up atomization regimes. The rich and complex multi-scale two-phase dynamics are characterized in terms of the fractal dimensions evaluated for 15,000 high-resolution images obtained from long-distance microscopy. For each flow condition, the measured probability density functions (PDF) of the fractal dimensions are compared to the corresponding Gaussian normal distribution and interpreted based on the higher order moments of statistics (Skewness and Kurtosis). A power-law correlation is proposed between the image-based fractal dimensions and the droplet sizing obtained from laser-diffraction measurements (Malvern, Spraytec). Total of 15 flow conditions are considered to generate a large database for the fractal analysis of the two-phase flow under a broad range of inertial (destabilizing) and viscous (stabilizing) effects.

**Figure 1:**
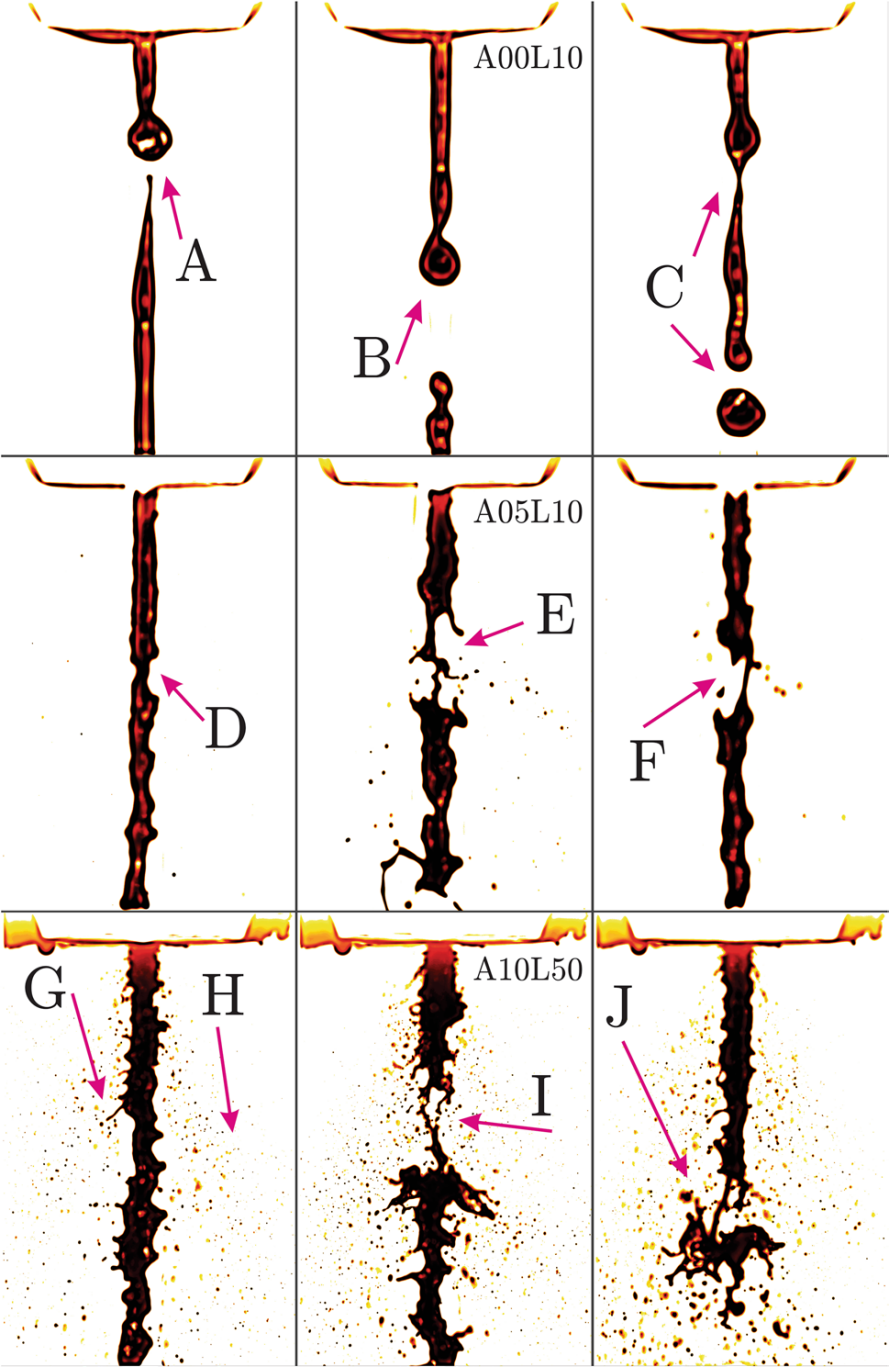
Bio-oil preheated to $T = 24$ $(^{\circ }$C) (*T*24 cases) with liquid $q_f = 4.5, 22.5$ (kg/h) (*L*10 and *L*50 cases at 10 and 50 (lb/h)) and air $q_a = 0, 5, 10$ (l/min) (*A*00, *A*05 and *A*10 cases) injection rates. (FOV: $9.25d_a \times 14.27d_a$).

**Figure 2:**
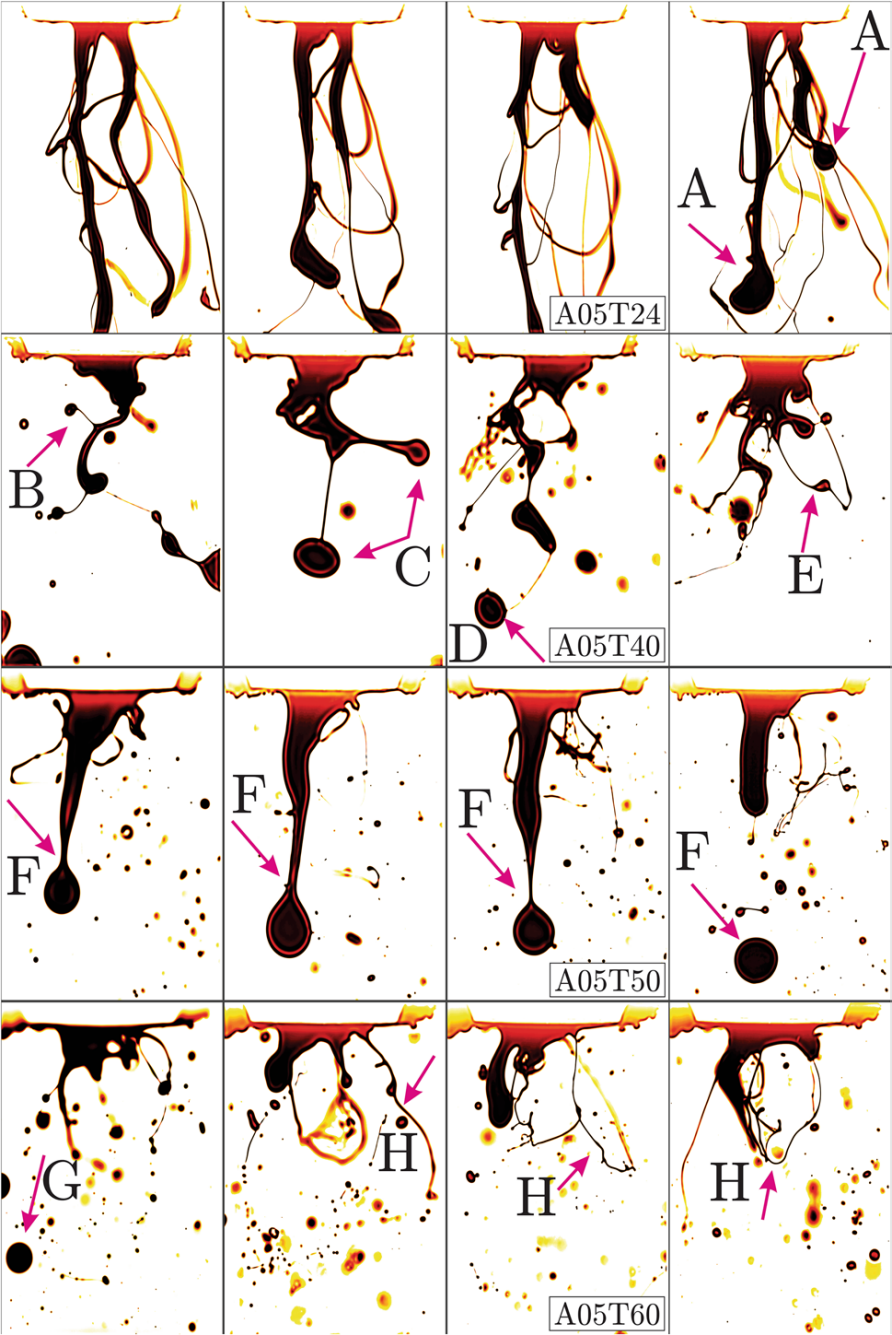
Bio-oil injected at the preheating temperatures $T = 24, 40, 50, 60$ $(^{\circ }$C) (*T*24, *T*40, *T*50 and *T*60 cases), liquid $q_f = 1.8$ (kg/h) (*L*04 cases at 4 (lb/h)) and air flow rates of $q_a = 5$ (l/min) (*A*05 cases). (FOV: $9.43d_a \times 14.15d_a$).

## Results

The presented results include three main categories. First, instantaneous snapshots obtained from the long-distance microscopy are provided to unveil the complex two-phase dynamics and instability mechanisms for 15 different flow conditions. Second, the corresponding fractal dimensions (*D*) are evaluated to generate a comprehensive dataset from analysing 15,000 high-resolution images. This large dataset enables us for a detailed statistical analysis in terms of probability density functions (PDF), mean values ($\overline{D}$) as well as higher order moments such as skewness (*S*) and kurtosis (*K*). The third category presents the results from laser diffraction droplet sizing for different flow conditions and a power-law correlation is developed between the fractal dimensions ($\overline{D}$) and the Suater mean diameter (SMD) for the global droplet size. To compare the competing effects among gas/liquid inertia, viscous diffusion, gravity and surface tension for different measurement cases, dimensionless groups (gas to liquid momentum flux ratio, liquid Reynolds number, gas Reynolds number, liquid Weber number, gas Weber number, Bond number and Ohnesorge number) are obtained and presented in in Table 2.

### Instability mechanisms shaping the two-phase pattern complexity

The first row in Fig. 1 shows the instantaneous snapshots of the pyrolysis bio-oil injected at the temperature of $T = 24$ $(^{\circ }$C) (*T*24 cases), liquid flow rate of $q_f = 4.5$ (kg/h) (*L*10 cases at 10 (lb/h)) and no air flow rate $q_a = 0$ (l/min) (*A*00 case). The field of view (FOV) size of the snapshots is given in the figure caption based on the air discharge diameter $d_a$. These *A*00*L*10 cases (see arrows A,B,C in Fig. 1) demonstrate a falling laminar liquid column going through symmetric contractions/expansions associated with the varicose mode of instability. The initially injected liquid, which is accelerated due to gravity, is classified beyond the dripping regime and falls into the jetting regime^36^. Growth of the perturbations due to the destabilizing effect of surface tension lead into the Savart–Rayleigh–Plateau (RP) instability^67–69^. The Young–Laplace relation suggests that the high-curvature contraction zones experience high pressures while the low-curvature expansion regions are associated with reduced local pressures^70^. This mechanism continuously induces internal liquid flows from the thinning high pressure zones to the thickening low pressure regions. Eventually, the thinnest liquid threads pinch-off leading into droplet formation (see arrows A,B,C). This mechanism is the nature’s ability to reduce the surface energy of a gas/liquid interface.

Introducing the atomizing air flow at $q_a = 5$ (l/min) (*A*05*L*10 cases) results in the formation of non-symmetric deformations due to the sinusoidal mode of instability (downstream of arrow D) as demonstrated in the second row of Fig. 1. At some time instants (shown by arrows E and F), the liquid column thinning leads into its local rupture and formation of ligaments and small droplets. A more extreme flow regime is displayed in the third row of Fig. 1, where both the liquid and air flow rate are significantly elevated to $q_f = 22.5$ (kg/h) (50 (lb/h)) and $q_a = 5$ (l/min) (*A*10*L*50 cases). In addition to the promoted short-wave (high frequency) sinusoidal jet meandering and the liquid column rupture, two new phenomena are observed. The first interesting phenomenon is the small local liquid ejections normal to the streamwise liquid column as pointed by arrow G. These local ejections are due to the Rayleigh–Taylor (RT) instability mechanism emerging as small patches of high-density liquid accelerating into the low-density gas phase. The local RT accelerations can also occur on top of the primary KH waves to form the ligaments further breaking up into smaller droplets^41^. With sufficient momentum, the ejecting patches of liquid detach from the main body of the liquid jet and reshape into droplets. Various levels of the gas/liquid inertia in Fig. 1, clearly reveal that the onset and growth of the instabilities dictate the level of complexity in two-phase geometrical patterns. The second phenomenon, marked by arrows H, is the appearance of numerous small size droplets stripped from the gas/liquid shear layer due to the large velocity differences causing the KH instability^40^. These stripped droplets could also be linked to the transition from initial linear KH instability to non-linear state and RT-type ligament formation on top of the primary KH vortex roll-ups at the gas/liquid interface^41^. As shown by arrow I, intense liquid column ruptures can also generate tiny ligaments and small droplets. Furthermore, droplet generation can also occur due to the leading tip vortex pinch-off (see arrow J) typically observed in transitioning/starting jets^26,71^.

**Figure 3:**
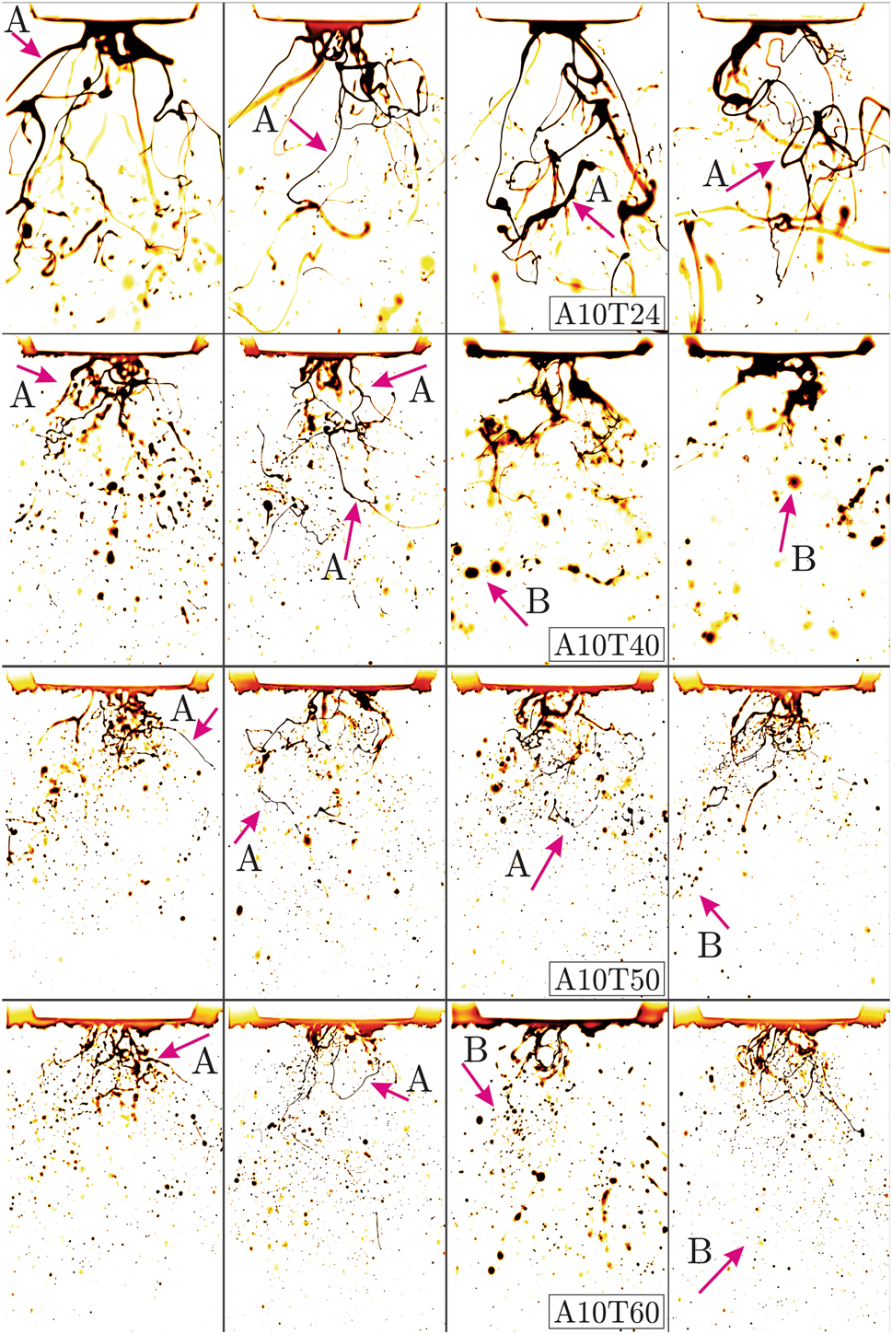
Bio-oil injected at the preheating temperatures $T = 24, 40, 50, 60$ $(^{\circ }$C) (*T*24, *T*40, *T*50 and *T*60), liquid flow rate of $q_f = 1.8$ (kg/h) (*L*04 cases at 4 (lb/h)) and air flow rate $q_a = 10$ (l/min) (*A*10 cases). (FOV: $9.43d_a \times 14.15d_a$).

**Figure 4:**
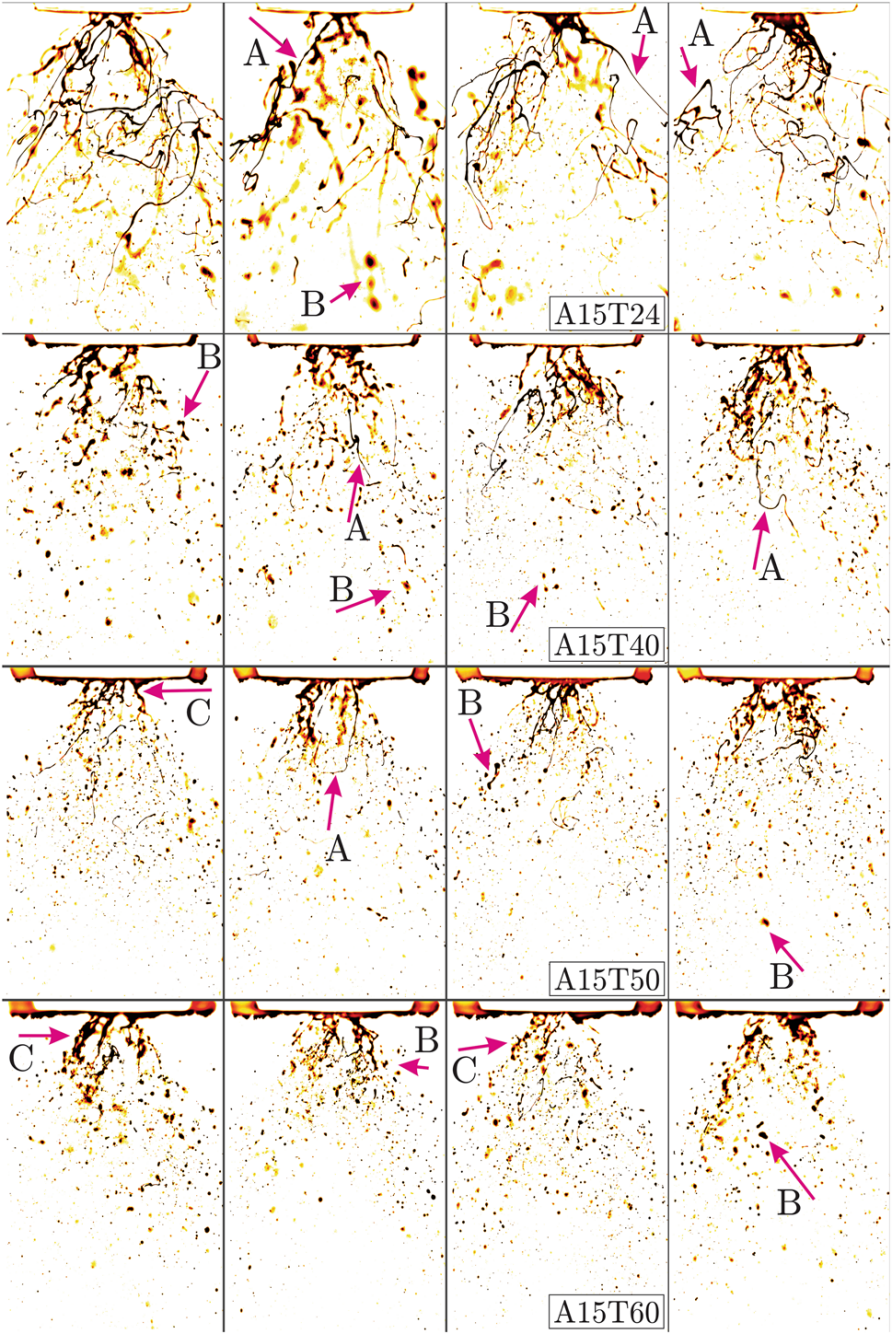
Bio-oil injected at the preheating temperatures of $T = 24, 40, 50, 60$ $(^{\circ }$C) (*T*24, *T*40, *T*50 and *T*60), liquid flow rate of $q_f = 1.8$ (kg/h) (*L*04 cases at 4 (lb/h)) and air flow rate $q_a = 15$ (l/min) (*A*15 cases). (FOV: $9.43d_a \times 14.15d_a$).

The resisting mechanism acting against the above-mentioned destabilizing inertial forces is through the viscous damping of the growing perturbations contributing to more stable conditions. In order to unveil the effect of viscosity on the two-phase geometrical/topological complexity, bio-oil is preheated to $T = 24, 40, 50, 60$ $(^{\circ }$C) and injected at $q_f = 1.8$ (kg/h) (*L*04 cases at 4 (lb/h)) as shown in Fig. 2. The first row exhibits the instantaneous snapshots of the bio-oil injection at the preheating temperature of $T = 24$ $(^{\circ }$C) and air flow rate of $q_a = 5$ (l/min) (*A*05*T*24 cases). The thick falling liquid stripes (pointed by the A arrows in Fig. 2) suggest a strong viscosity dominance over the gas/liquid inertia. As a result, the short wave-length (high frequency) perturbations are suppressed and the purely large wave-length oscillations (meandering) produce no droplets. Elevated preheating to $T = 40$ ($^{\circ }$C) (second row in Fig. 2) remarkably alters the complexity of the emerging liquid column showing fascinating bifurcations (pointed by arrows B and C) into pairs of diverging ligaments. As in fractals, the bifurcation mechanism cascades down into smaller ligament pairs accelerating into the low-density gas and produce a few droplets. These accelerating ligaments are another example of the RT instability which can produce droplets at larger scales comparable to that shown by arrow D. It is also observed that thinner liquid threads can produce pearl-type droplets as pointed by arrow E. For the $T = 50$ $(^{\circ }$C) case shown in the third row of Fig. 2, number of large/small droplets increase while simultaneously the RP droplet pinch-off shown by F arrows is taking place. Eventually at $T = 60$ $(^{\circ }$C) in the fourth row, reduced viscous effects are dominated by inertia and result in small droplets, short ligaments and thin liquid sheets pointed by the G and H arrows. In Fig. 3, bio-oil is injected at $q_f = 1.8$ (kg/h) (4 (lb/h)) and preheated to $T = 24, 40, 50, 60$ $(^{\circ }$C) while the atomizing air flow is fixed at $q_a = 10$ (l/min). Due to stronger gas phase inertial forces, length scales become smaller and narrow distorted structures are shaped. Accordingly in the first row, the strong viscous oil at $T = 24$ $(^{\circ }$C) remarkably defies breaking up to form extremely thin, elongated and tangled liquids threads (ligaments) as shown by A arrows. With elevated preheating to $T = 40$ ($^{\circ }$C) (second row of Fig. 3), the thin ligaments are shortened due to earlier break-up, hence more small droplets are produced as shown by B arrows. Similarly droplet formation is promoted when increasing the bio-oil preheating temperatures to $T = 50$ and $T = 60$ $(^{\circ }$C) in third and fourth rows, respectively. Further enhancing the gas phase inertia to $q_a = 15$ (l/min) (Fig. 4) reveals promoted atomization and the thin ligaments (A arrows) and liquid sheets (C arrows) are quickly replaced by small droplet (B arrows) formation. Similar to earlier cases, oil preheating accelerates the transition from jet break-up to fully atomized spray regime.

Above observations from high-resolution microscopy nicely reveal the transitioning regimes for a wide range of flow conditions. It is found that the combination and/or the competition between variety of instability mechanisms produces rich phenomena and diversified complex geometries. From an instability point of view, increased geometrical intricacy can be associated with a transition from larger wavelengths (low frequency) into a flow regime comprised of the spatio-temporal evolution of the wave groups exhibiting a broad spectrum of small-to-large wavelengths (frequencies). As visualized above, variable morphologies shaped under different flow conditions are basically the geometrical patterns arising from instability mechanisms as well as their corresponding vortex dynamics. Different observations of the local/global instabilities can be interpreted in terms of the wavelength distribution on the liquid surface, jet meandering, KH vortex roll-up or RT-shaped ligament distributions. In the following section, the dominant fractal character of the statistics can be correlated back to the above visualized shape of wavelength distribution on liquid jets, or the shape of ligament distributions.

### Higher-order statistics of fractal dimensions

**Figure 5:**
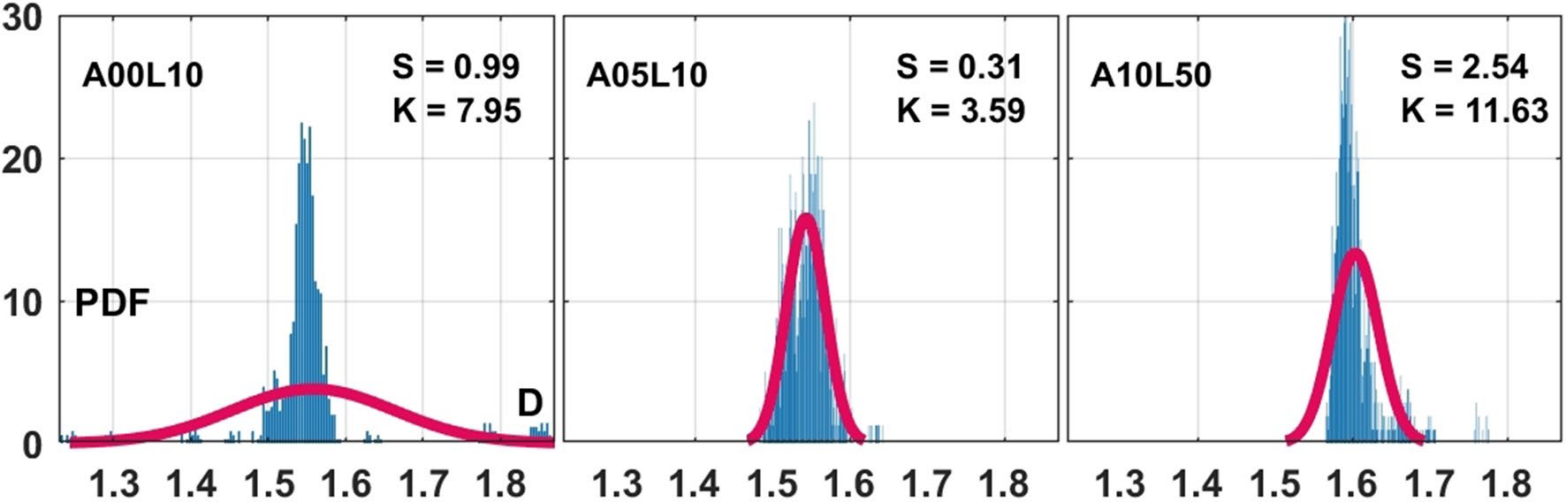
Discrete (blue bars) probability density function (PDF) of the fractal dimension *D* for the *A*00*L*10, *A*05*L*10 and *A*10*L*50 cases at bio-oil preheating temperature of $T = 24$ $(^{\circ }$C) (*T*24 cases) compared to the Gaussian normal distribution (red line), where *S* and *K* respectively denote skewness and kurtosis.

**Figure 6:**
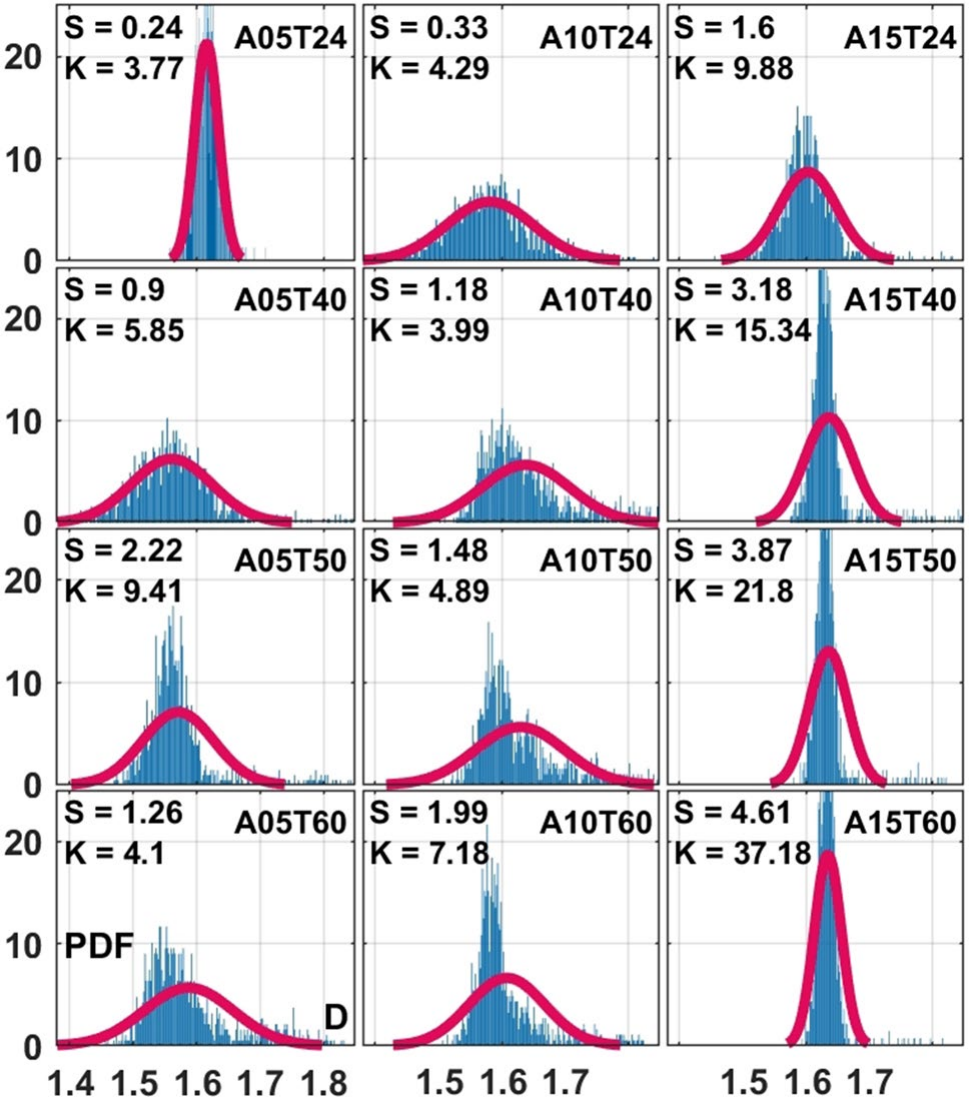
Discrete (blue bars) probability density function (PDF) of the fractal dimension *D* compared to the Gaussian normal distribution (red line), where *S* and *K* respectively denote skewness and kurtosis. Liquid flow rate: $q_f = 1.8$ (kg/h) (*L*04 cases at 4 (lb/h)); bio-oil preheating temperatures: $T = 24, 40, 50, 60$ $(^{\circ }$C) (*T*24, *T*40, *T*50 and *T*60 cases); Air flow rates: $q_a = 5, 10, 15$ (l/min) (*A*05, *A*10 and *A*15 cases).

In order to quantify the geometrical complexities observed in the 15 different two-phase flow conditions visualized above, the corresponding fractal dimensions (*D*) are evaluated using a box-counting^72,73^ method to create a comprehensive dataset from analysing 15,000 high-resolution images (1000 images per flow condition). The large dataset produced is then used for a thorough statistical analysis. As also reported in earlier studies^74^, some of the visualized liquid fragments are highly three-dimensional and from a single viewing angle, presumably information is lost. However, this is accounted for due to the large number of statistics produced in the present study. As a result, the discrete probability density functions (PDF) are obtained from 1000 samples per case and compared to the corresponding normal Gaussian distribution. The higher order moments of statistics are also evaluated based on the data skewness (*S*) and kurtosis (*K*). In the following, mean fractal dimension values are denoted with an over-line as $\overline{D}$. Statistical distribution of the fractal dimensions associated with instantaneous two-phase geometries (similar to those shown in Fig. 1) are presented in Fig. 5. In the vertical coordinate (both Figs. 5, 6), the frequency count (the number of times the fractal dimension appears) are given, while these values are normalized by the area under the plots to imply probability density functions (PDF). Therefore, the area under the PDFs are unity as should be the sum of all possibilities of events. The corresponding Gaussian distribution is obtained using the mean and variance calculated from the 1000 data samples for each case shown. Considering uncertainties from various levels of image resolution, earlier studies suggest that the spray fractal dimensions may vary from lower Weber number (e.g. 2nd wind-induced atomization $D = 1.25$) regimes^75,76^ to higher values associated with turbulent gas jets, shear layers and mixing layers $(D = 1.45-1.6)$^27,28,75^. The geometrical complexity also varies instantaneously due to the chaotic/intermittent nature of the gas/liquid interface reshaping.

In Fig. 5, the discrete PDF is represented by the blue vertical bars and compared with their corresponding Gaussian normal distribution (red line). For a normal distribution, $S = 0$ and $K = 3$ respectively imply that the data is symmetric and has neither thick (heavy) nor thin (light) tails. For instance, the discrete PDF in *A*00*L*10 case with $S = 0.99$ is not too far from symmetry. However, it deviates from a normal distribution due to its rather heavy tails at kurtosis value of $K = 7.95$. Such a distribution in geometrical complexity is in accordance with the formation of mainly mono-dispersed droplet sizes (sharp PDF peak) and a few satellite droplets (the PDF tails), in a way consistent with our understanding of the RP instability mechanism^39^. While the statistical distribution is found concentrated near the 1.5–1.6 values, it demonstrates long tails due to large Kurtosis value of $K = 7.95$. These long tails show that there are a few dimension values very close to either 1 or 2. As shown in the sample snapshots, the droplet pinch-off distorts the rather straight falling liquid column into complex structures resulting in reasonable dimension values statistically close to 1.5–1.6. On the other hand, introducing the air flow inertia in the *A*05*L*10 case, produces a relatively normal distribution with $S = 0.31$ and $K = 3.59$. For a more extreme flow condition of the *A*10*L*50 case, the right tail becomes longer and the distribution is positively skewed with $S = 2.54$. Large kurtosis ($K = 11.63$) compared to the Gaussian distribution, forms a very strong peak which rapidly decays to heaver tails. Figure 5 clearly suggests that a proper fractal analysts in chaotic and irregular fluid flows should not be limited to single- or mean-values, but rather interpreted by a thorough statistical analysis. The higher order statistics reveal the random, anisotropic and chaotic nature of the instantaneous complex reshaping in two-phase flows subject to different inertial destabilizations.

In addition to the inertial destabilizations, the stabilizing viscous effects on the distribution of the fractal complexities are explored. The rows in Fig. 6 are associated with the viscous effects by oil preheating to $T = 24, 40, 50, 60$ $(^{\circ }$C) (*T*24, *T*40, *T*50, *T*60 cases) while the columns demonstrate gas phase inertial effects (*A*05, *A*10, *A*15 cases) and the liquid flow rate is maintained at $q_f = 1.8$ (kg/h) (*L*04 cases at 4 (lb/h)). One interesting general observation is that the skewness values are all positive, suggesting that sudden occurrence of certain physical mechanisms always result in increased geometrical complexity while the opposite is not true. Some examples of such mechanism are when consistently meandering liquid columns, oscillating thin ligaments or stretching liquid sheets suddenly burst into small-scale structures with more complex geometries. The *A*05*T*24 case is the only distribution in Fig. 6 showing a rather normal Gaussian behaviour. With increased preheating temperatures (reduced viscous damping) and/or air flow inertia, distribution of the fractal dimensions deviate from the Gaussian symmetry. This is due to the higher probability of the instability mechanisms to grow into chaotic sudden distortions towards more intricate flow structures. Another interesting finding is that the kurtosis values are significantly larger for the *A*15 cases in which the atomizing air flow inertia is the highest. This suggests higher probability of finding fractal dimension values away from the mean (larger or smaller). Accordingly, the large kurtosis values imply intermittent variation of the geometrical complexity rather than exhibiting orderly reshaping in time.

### Correlation between the fractal complexity and global droplet size

**Figure 7:**
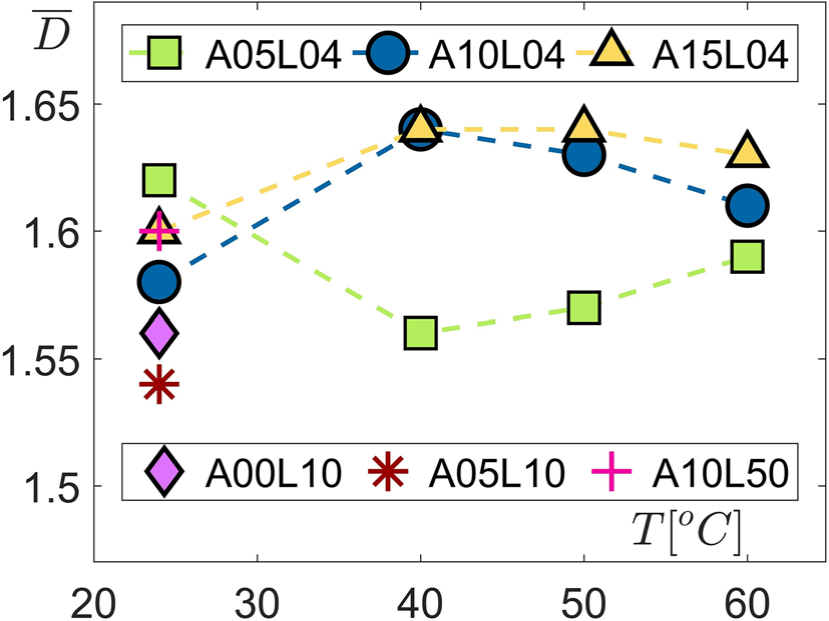
Averaged fractal dimensions $\overline{D}$ compared for 15 different two-phase flow conditions imposed by varying the liquid flow rates (*L*04, *L*10 and *L*50 cases), bio-oil preheating temperatures (*T*24, *T*40, *T*50 and *T*60 cases) and air flow rates (*A*05, *A*10 and *A*15 cases).

Results presented in the previous section suggest that a proper analysis of the geometrical complexity in two-phase flows is best achieved by analysing the statistical distribution of the fractal dimensions. However, the averaged value $\overline{D}$ can provide some overall information to be used for comparison among different cases. Further, the mean values of the fractal dimensions can be used to obtain a correlation with a global droplet size such as Suater mean diameter (SMD) obtained from laser diffraction measurements. Figure 7 presents the mean fractal dimension values ($\overline{D}$) averaged over 1000 samples for each of the 15 flow cases. The most interesting observation is the different behaviour observed for the *A*05*L*04 cases with increased temperature, while the *A*10*L*04 and *A*15*L*04 cases exhibit a similar trend. Such a phenomenon is often observed in laminar to turbulent transition where flow properties demonstrate similarity beyond certain Reynolds numbers. Here, the geometrical complexity of the two-phase flow demonstrates a similar behaviour with elevated inertial effects. When preheated to $T = 40, 50, 60$ $(^{\circ }$C), the *A*10*L*04 and *A*15*L*04 cases attain larger values of the mean fractal dimension compared to the *A*05*L*04 cases as a measure of their more complex structure.

**Figure 8:**
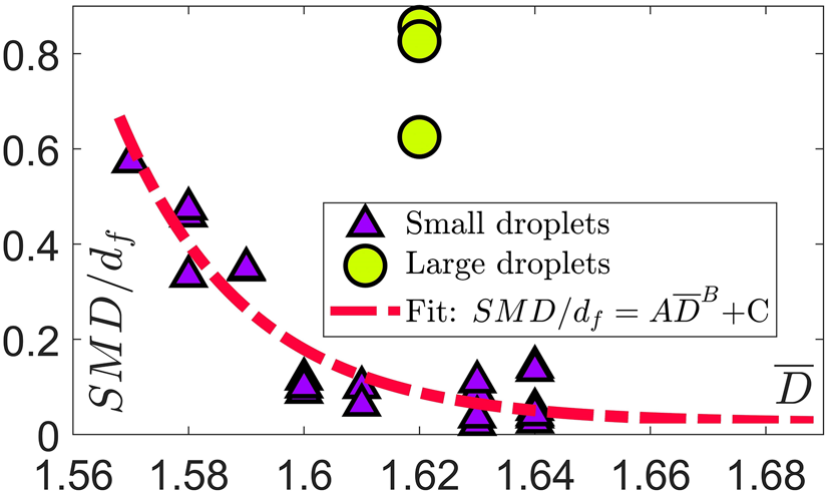
Decoupling in small (triangles) and large (circles) droplets represented by Suater mean diameter (SMD) as a global size. The “small” droplets belong to a class following a power-law correlation, while the “large” droplets behave independently. The power-law correlation $SMD/d_{f} = A\overline{D}\,^{B} + C$ between the averaged fractal dimensions $\overline{D}$ and SMD ($d_{f}$: liquid discharge diameter) with coefficients $A = 3.327 \times 10^{13}, \, B = -70.2, \, C = 0.0229$ is represented by the red line.

Except for very few applications, the ultimate goal of the liquid injection is to produce fully atomized sprays where small scale droplets can enhance heat/mass transport. The global fineness of the droplets in two-phase flow systems can be defined based on the overall liquid volume to surface ratio characterised by the Suater mean diameter (SMD). As a representative, the centreline SMD values 10 *mm* downstream of the injection orifice are considered. With increased interest in image classification techniques, fractal dimensions can be used to correlate a certain instantaneous image with a representative droplet size such as SMD. Therefore, a power-law correlation is developed between the mean fractal dimensions and SMD values for different flow conditions. As shown in Fig. 8, the correlation coefficients ($A = 3.327 \times 10^{13}, \, B = -70.2, \, C = 0.0229$) are found by applying the method of least-squares to fit a power-law function of the form $SMD/d_{f} = A\overline{D}\,^{B} + C$ to the discrete measured data points where $d_{f}$ denotes liquid discharge diameter. A very interesting finding is the “decoupling” between the large and small droplets. Here, the “small” droplets class nicely follow the power-law correlation, while the “large” droplets beyond a level behave independently. It can be seen that the global droplet size is reduced as the mean fractal dimension increases. This is in accordance with our visualizations where different instability mechanisms take over the two-phase break-up physics and produce more complex geometries. In other words, increased geometrical complexity can be associated with enhanced atomization and formation of smaller droplets. On the other hand, the averaged fractal dimension values seem to exhibit insensitivity to large droplets. While 2D spray images are used for the fractal analysis, incorporating the out of focus liquid structures and large number of statistics provide a good representation of the 3D spray. It should be noted that the proposed correlation is most accurate for the range of parameters considered in the present study with an attempt to cover a broad range of two-phase flow regimes.

## Discussion

Similar to that of various phenomena ubiquitously taking place in nature, fluid flows exhibit fascinatingly complex geometrical reshaping. Such geometrical intricacies are particularly remarkable in two-phase interfacial flows. In the present study, fractal analysis is conducted to characterize the random and irregular geometrical structures formed during the bio-oil break-up and atomization. High-resolution long-distance microscopy imaging is carried out to obtain 15,000 snapshots of the two-phase interactions for 15 different flow conditions during the air-assisted atomization of pyrolysis oils. For all the high-resolution images, fractal dimensions are evaluated to characterize the complex multi-scale two-phase dynamics. In addition, droplet sizing is carried out using laser-diffraction measurements (Malvern’s Spraytec). Results are discussed in three main categories:

- Through careful analysis of instantaneous high-resolution snapshots, it is found that the geometrical complexity of the two-phase flow structures evolve in accordance with the onset of linear instability mechanisms and their non-linear growth. The competing effect between the destabilizing inertial forces, surface tension and the stabilizing (damping) fluid viscous forces govern the underlying two-phase physics. The competing instability mechanisms such as Kelvin–Helmholtz (KH), Rayleigh–Taylor (RT) and Rayleigh–Plateau (RP), which govern the two-phase dynamics, lead into the formation of very diversified geometrical shapes at different irregularity levels and length scales. While the 1000 instantaneous liquid structure for each of the 15 flow cases are very unlikely to be identical, each case represents a class of geometrical pattern. Under variable levels of gas/liquid inertial forces acting against different viscous resistance scenarios, geometrical complexities of liquid break-up are altered in accordance with the formation of meandering liquid columns, thick falling liquid stripes, stretched/distorted narrow liquid threads, oscillating ligaments, liquid sheets and deformed droplets which all appear within a broad range of length scales and shapes often dictated by the local vortex dynamics.
- Fractal dimensions are evaluated for all the 15,000 snapshots to characterize the visualized geometrical complexity of the liquid break-up. Even though this is a cumbersome box-counting task to evaluate the fractal dimensions for large number of high-resolution images, the output is a comprehensive set of data to conduct a thorough statistical analysis. The discrete probability density function (PDF) associated with the temporal variation of fractal dimensions reveal the complexity of reshaping two-phase structures. Compared to their corresponding Gaussian normal PDFs, the discrete distributions are found to behave differently for most of the flow cases. This is verified by calculating the higher-order moments of statistics, skewness and kurtosis. The skewness values suggest that the discrete PDFs are mostly non-symmetric with respect to the mean fractal dimensions. Interestingly, all the discrete PDFs are positively skewed (longer right tail) suggesting that during the regular/organized geometrical reshaping there may be some physical mechanisms abruptly taking place. Examples of such sudden distortions are liquid column rupture, ligament and sheet bursting and local liquid accelerations all of which result in increased geometrical complexity, hence larger fractal dimensions. At increased preheating temperatures (reduced viscosity) and/or inertial forces, where perturbations grow into chaotic sudden distortions, fractal dimension statistics tend to become more skewed and complexity increases. At the highest air flow inertia (*A*15 cases), it is found that the kurtosis values become significantly larger. This remarkable finding can be attributed to the intermittent temporal fluctuations of the geometrical complexity. This means that there is a higher probability of finding fractal dimension values either larger or smaller than mean values. Therefore, orderly temporal reshaping is replaced by chaotic geometrical deformations.
- Comparing the averaged fractal dimensions, i.e. the geometrical features, among all the 15 different test conditions demonstrate a behaviour similar to that of laminar to turbulent transition in which the flow quantities beyond the transition exhibit similar trends that are found different from that of the flow conditions below the transition regime. Droplet sizes obtained from laser diffraction measurements represent the overall liquid volume to surface ratio by a global value known as the Suater mean diameter (SMD). Within the range of the present measurements an interesting decoupling appears between the large and small droplets where the SMD values are classified based on mean fractal dimensions. The smaller droplets class nicely follows a power-law correlation of the form $SMD/d_{f} = A\overline{D}\,^{B} + C$ where coefficients ($A = 3.327 \times 10^{13}, \, B = -70.2, \, C = 0.0229$) are optimized using the method of least-squares. The proposed correlation verifies our microscopic imaging in which the break-up and atomization mechanisms cascade down to smaller length scales as the two-phase geometrical complexity increases. On the other hand, a class of larger droplets are found for which the fractal complexity remains relatively insensitive.

**Table 1:** Physical properties and test conditions.

Oil properties	$\rho _{f}$ (kg/m$^3$)	$\sigma$ (mN/m)			$\nu _{f}$ mm${^2}$/s)		
	${(20\,^{\circ }{\text {C}})}$	${(20\,^{\circ }{\text {C}})}$	${(40 \,^{\circ }{\text {C}})}$	${(60 \,^{\circ }{\text {C}})}$	${(20 \,^{\circ }{\text {C}})}$	${(40 \,^{\circ }{\text {C}})}$	${(60 \,^{\circ }{\text {C}})}$
	1217	40.0	38.5	37.2	227.2	65.1	24.7

Test conditions	Air flow rate	Liquid flow rate			Preheating temperature		
	$q_a$ (l/min)	$q_f$ (kg/h)			*T* $(^{\circ }$C)		
	5, 10, 15	1.8, 4.5, 22.5			24, 40, 50, 60		

Density, surface tension^77^ and kinematic viscosity are respectively denoted as $\rho _{f}$, $\sigma$ and $\nu _{f}$.

**Figure 9:**
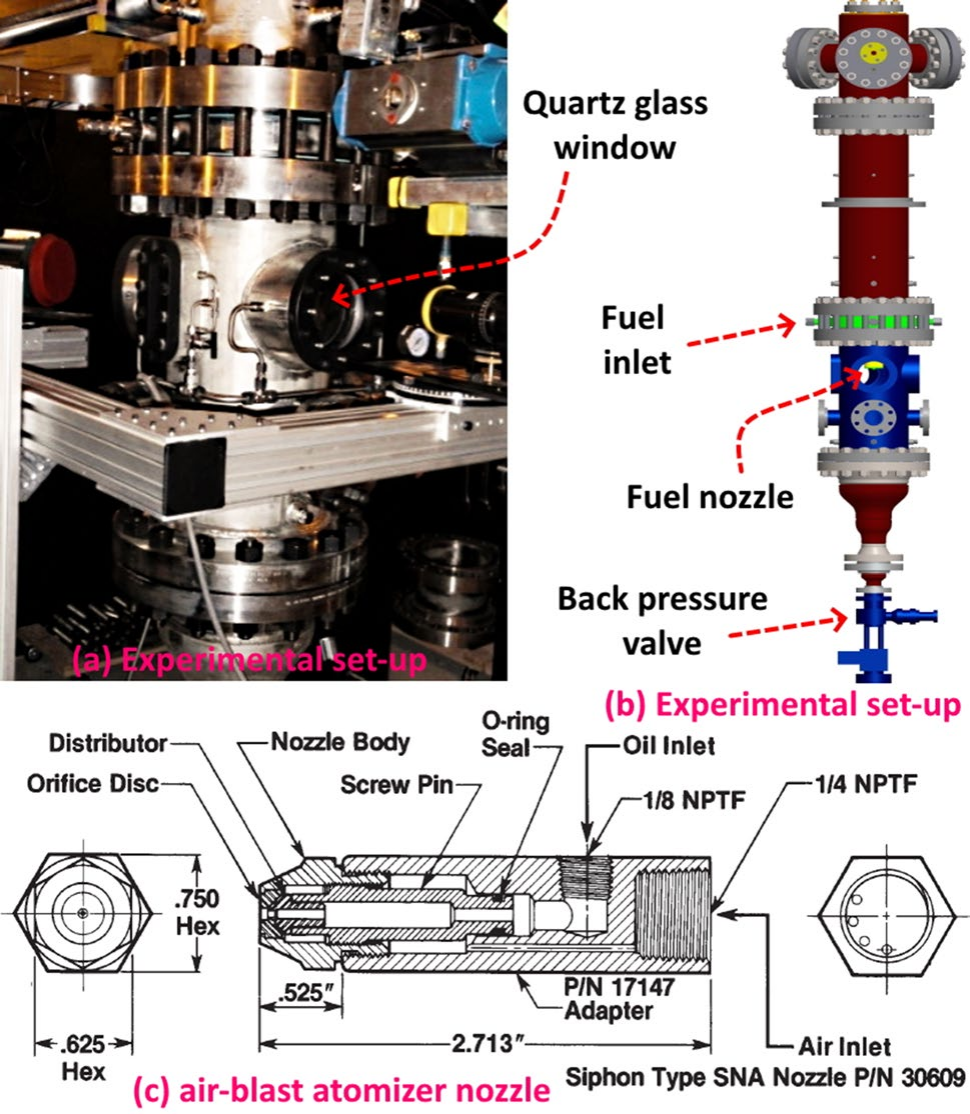
Experimental set-up (**a**,**b**) and air-blast atomizer nozzle geometry (**c**).

## Methods

### Experiments

Figure 9 shows the experimental set-up for spray test facility and nozzle geometry. The bio-oil jet is issued through an air-blast atomizer (siphon type SNA nozzle, $$P/N \, - 30609$$)^78^ with liquid discharge area of $$A_f = 0.937$$   (mm$$^2)$$ (equivalent diameter $$d_f = 1.092 \, $$ (mm)) and air flow cross-section of $$A_a = 4.997$$   (mm$$^2)$$ (equivalent diameter $$d_a = 2.523$$   (mm)). Liquid is injected with a co-flowing air stream of the given flow rate, into the otherwise quiescent air within the optically accessible constant volume chamber at atmospheric pressure and room temperature. Bio-oil flow rates of $$q_f = 1.8$$, 4.5 and 22.5 (kg/h) (L cases at 4, 10 and 50 (lb/h)), atomizing air flow rates (A cases) at $$q_a = 5, 10, 15$$ (l/min) and oil preheating temperatures (T cases) of $$T =$$ 24–60 $$(^{\circ }$$C) are considered. Physical properties and test conditions are given in Table 1 where density, surface tension^77^ and kinematic viscosity are respectively denoted as $$\rho _{f}$$, $$\sigma $$ and $$\nu _{f}$$. The dimensionless groups obtained for different flow test conditions and variable physical properties are given in Table 2 where $$q = \rho _{a}{U_{a}}^2/\rho _{f}{U_{f}}^2$$, $$Re_{f} = d_{f}U_{f}/\nu _{f}$$, $$Re_{a} = d_{f}U_{a}/\nu _{a}$$, $$We_{f} = \rho _{f}{U_{f}}^2d_{f}/\sigma $$, $$We_{a} = \rho _{a}{U_{a}}^2d_{f}/\sigma $$, $$Bo = g(\rho _{f}-\rho _{a}){d_{f}}^2/\sigma $$ and $$Oh_{f} = \sqrt{We_{f}}/Re_{f}$$ respectively denote gas to liquid momentum flux ratio, liquid Reynolds number, gas Reynolds number, liquid Weber number, gas Weber number, Bond number and Ohnesorge number. These dimensionless numbers demonstrate the competing effects among gas/liquid inertia, viscous diffusion, gravity and surface tension for different measurement cases as well as the effects due to preheating temperatures.

**Table 2:** Dimensionless groups obtained for different flow test conditions and variable physical properties.

Cases	*q*	$Re_{f}$	$Re_{g}$	$We_{f}$	$We_{g}$	*Bo*	$Oh_{f}$
*A*00*L*10	0	5.33	0	41	0	0.354	1.20
*A*05*L*10	6.31	5.33	6309	41	258	0.354	1.20
*A*10*L*50	1.01	26.62	12,617	1021	1033	0.354	1.20
*A*05*T*24	39.72	2.12	6309	6.50	258	0.354	1.20
*A*05*T*40	37.68	7.41	5753	6.75	254	0.368	0.35
*A*05*T*50	36.54	12.63	5444	6.88	252	0.375	0.21
*A*05*T*60	35.44	19.54	5161	6.99	248	0.381	0.14
*A*10*T*24	158.87	2.12	12,617	6.50	1033	0.354	1.20
*A*10*T*40	150.72	7.41	11,506	6.75	1018	0.368	0.35
*A*10*T*50	146.17	12.63	10,888	6.88	1006	0.375	0.21
*A*10*T*60	141.76	19.54	10,323	6.99	991	0.381	0.14
*A*15*T*24	357.47	2.12	18,926	6.50	2324	0.354	1.20
*A*15*T*40	339.11	7.41	17,259	6.75	2290	0.368	0.35
*A*15*T*50	328.88	12.63	16,333	6.88	2264	0.375	0.21
*A*15*T*60	318.95	19.54	15,484	6.99	2229	0.381	0.14

### Long-distance microscopy and laser diffraction droplet sizing

Fifteen cases of pyrolysis bio-oil break-up regimes down to the fully atomized sprays are characterized using the high-resolution long distance microscopic imaging by generating a pulsed back-lighting illumination. Image acquisition is conducted using the 14-bit IMAGER PRO X 2M charge-coupled device (CCD) camera with CCD pixel size $7.4\,\upmu$m, CCD chip size $1600 \times 1200$ and read-out noise $< 12 e^-$. LaVision’s DaVis software is used for camera and image acquisition control. The camera is equipped with a Questar $QM-100$ long distance microscope lens to allow for resolving the smallest scales of liquid structure. The long distance microscope lens has a 15–35 *cm* working range with clear aperture of 63 mm and magnification up to $34\times$. A total of 15,000 images (1000 per case) are acquired at $1600 \times 1200$ pixels corresponding to the spatial resolution of 94.2 pixels/*mm*. In addition, laser diffraction droplet size measurements are carried out using Malvern’s Spraytec. The global fineness of the bio-oil break-up and atomization is represented by the Sauter Mean Diameter (SMD) which is a measure of liquid overall volume to surface ratio.

### Image processing and fractal analysis

**Figure 10:**
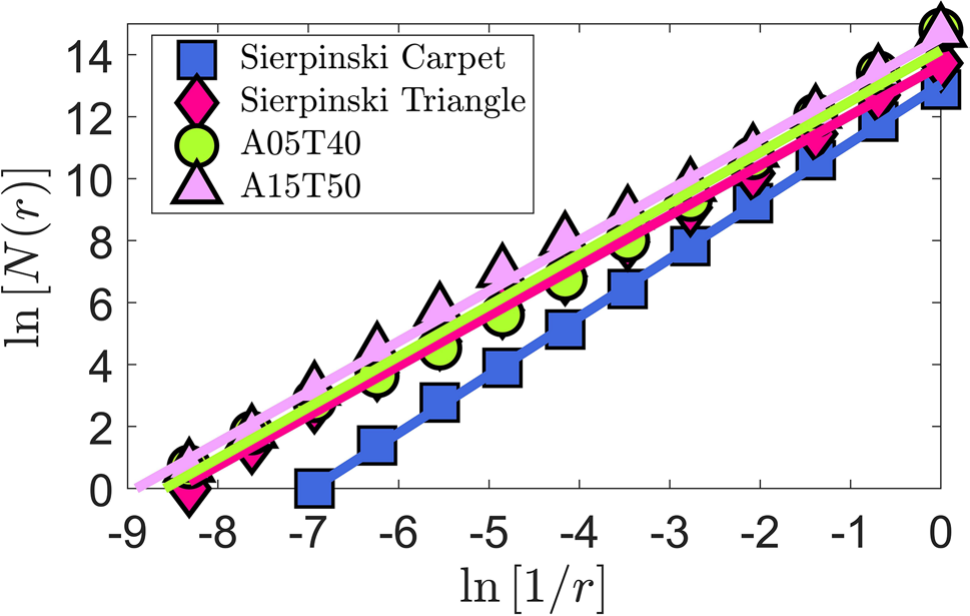
Linear fit obtained from the present box-counting^72,73^ algorithm to determine the fractal dimensions for the Sierpinski triangle and Sierpinski carpet^73^ compared to sample spray cases *A*05*T*40 and *A*15*T*50.

Fractal dimensions (*D*) are evaluated by successively refining the grid size and extracting the cells representing the complexity of the two-phase flow structure, the so-called “box-counting” algorithm^72,73^. For each of the 15 flow cases, fractal dimension statistics are evaluated by analysing 1000 high-resolution snapshots. A systematic approach is taken to pre-process the images before they can be properly used for fractal analysis. All the RGB images are first converted into gray-scale and normalised to represent liquid fraction. Five images are taken from the background light source to obtain the average back light intensity. The averaged field is then subtracted form all the instantaneous images. In order to correct for the out of focus liquid structures images are slightly de-blurred and then binarized with zero pixels in the background. To start the box-counting algorithm, images are padded into a pixel dimension at a power of 2. The box size *r* is initialized equal to the image size. Then, number of boxes of size *r* containing a minimum of one liquid pixel are counted as *N*(*r*). This process is continuously repeated by setting $r = \frac{r}{2}$ as long as $r > 1$. The resulting logarithmic data points ($\ln {N(r)} \, vs. \, \ln {(1/r)}$) are fitted to a linear function using the method of least squares. As shown in Fig. 10, the linear fits nicely represent the data points evaluated for the two test cases of the Sierpinski triangle and Sierpinski carpet compared to the sample spray cases *A*05*T*40 and *A*15*T*50. The slope of these lines gives the fractal dimension (*D*) of the image.

**Figure 11:**
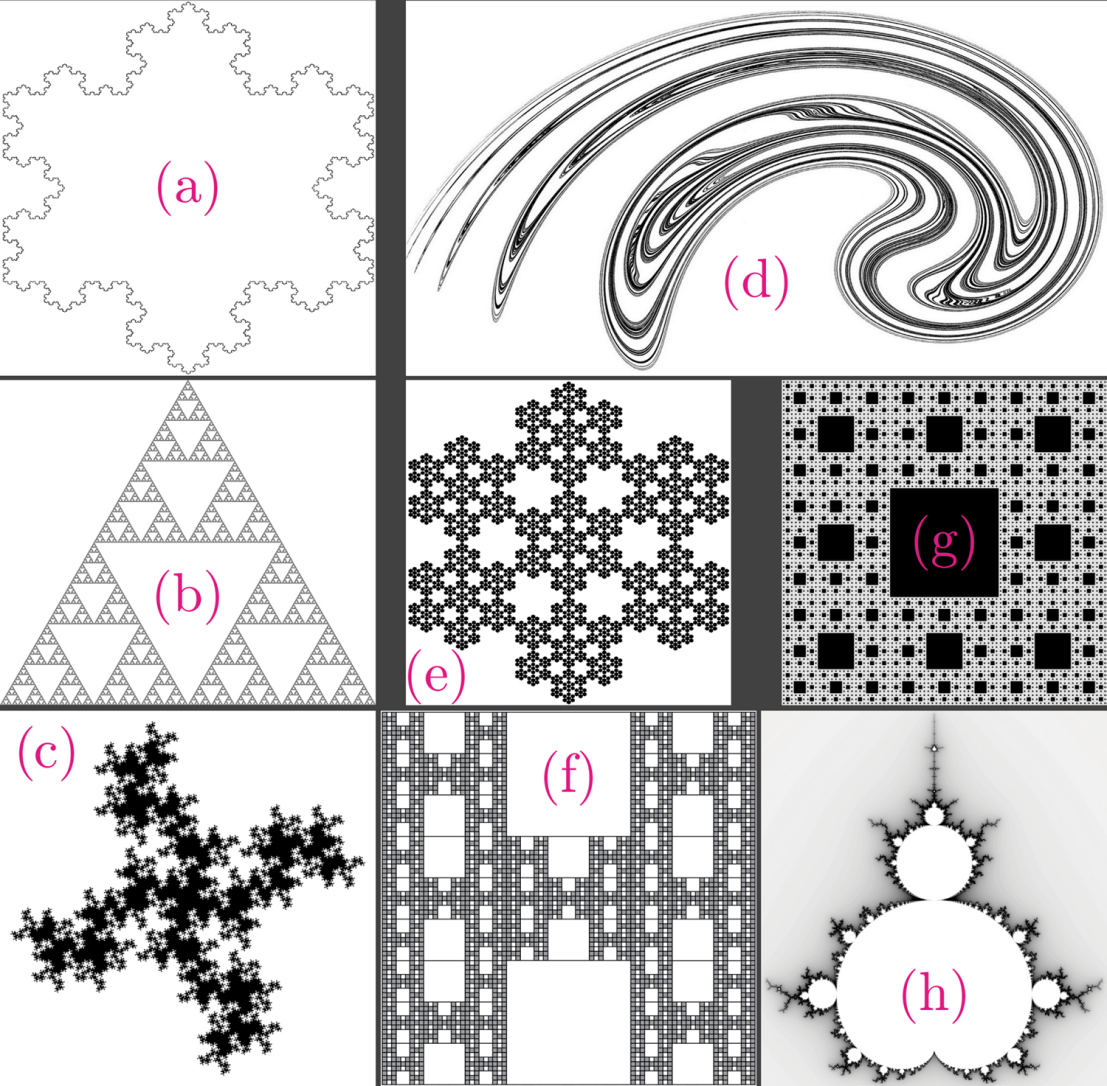
Fractal structures with known Hausdorff dimensions. See Table 3 for detailed numerical values and error estimation of the (**a**)–(**h**) patterns.

In order to verify the present box-counting approach, Fig. 11 demonstrates a set of geometrical structures with known Hausdorff dimensions. Fractal dimension values from the present box-counting compared to their corresponding Hausdorff dimensions and the error estimation are summarized in Table 3. It can be seen that the present study robustly evaluates the fractal dimensions of geometrically complex structures. Since nearly all of the fractal dimension values are slightly underestimated, the accuracy could be further improved by using higher resolution images. These geometrical patterns are carefully selected to cover a range of fractal dimensions expected from the present measurements. In addition, these benchmark structures exhibit diverse shapes and patterns suitable for testing the present algorithm.

**Table 3:** Fractal dimensions from the present box-counting^72,73^ compared to the corresponding Hausdorff dimensions of known geometrical patterns^73^ shown in Fig. 11.

Known fractals	Hausdorff dimension	Present box-counting	Error ($\%$)
(a) von Kock snow flake	1.262	1.298	2.84
(b) Sierpinski triangle	1.585	1.572	$-$ 0.85
(c) 32 Segment $\frac{1}{8}$-scale quadratic	1.667	1.656	$-$ 0.62
(d) Ikeda map	1.680	1.648	$-$ 1.89
(e) Hexa-flake	1.771	1.705	$-$ 3.73
(f) $H-I$ de Rivera	1.771	1.752	$-$ 1.09
(g) Sierpinski carpet	1.893	1.876	$-$ 0.91
(h) Mandelbrot set	2	1.910	$-$ 4.5

**Figure 12:**
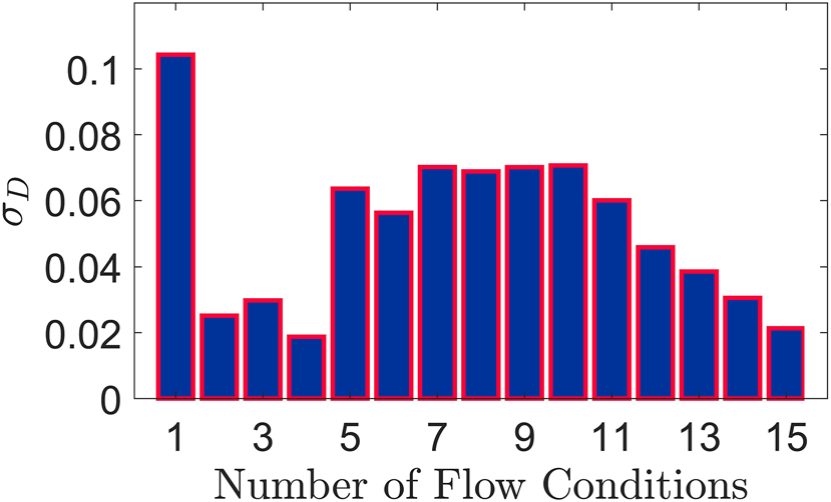
Standard deviation $(\sigma _D)$ of the fractal dimensions evaluated using 1000 samples for each of the 15 flow test conditions.

In order to ensure that the present measurements are repeatable and produce reasonably low errors, standard deviation $\sigma _D$ values associated with the evaluated fractal dimensions are determined. For each of the 15 flow test conditions, the corresponding standard deviation is evaluated for the fractal dimensions obtained from 1000 snapshots. As shown in Fig. 12, the standard deviation for most of the measurement cases can be approximated within the range 0.02–0.07. This is an indication of the precise measurements and accurate calculations of the fractal dimensions.
